# Genome-wide gene expression analyses reveal unique cellular characteristics related to the amenability of HPC/HSCs into high-quality induced pluripotent stem cells

**DOI:** 10.1186/s13287-016-0298-z

**Published:** 2016-03-15

**Authors:** Shuai Gao, Li Tao, Xinfeng Hou, Zijian Xu, Wenqiang Liu, Kun Zhao, Mingyue Guo, Hong Wang, Tao Cai, Jianhui Tian, Shaorong Gao, Gang Chang

**Affiliations:** Institute of Molecular Medicine, Health Science Center, Shenzhen University, Shenzhen 518060, China; National Institute of Biological Sciences, NIBS, Beijing 102206, China; Translational Medical Center for Stem Cell Therapy, Shanghai East Hospital, School of Medicine, Tongji University, Shanghai 200120, China; Ministry of Agriculture Key Laboratory of Animal Genetics, Breeding and Reproduction; National Engineering Laboratory for Animal Breeding; College of Animal Sciences and Technology, China Agricultural University, Beijing 100193, China; School of Life Sciences and Technology, Tongji University, Shanghai 200092, China

**Keywords:** Hematopoietic progenitor and stem cells, Induced pluripotent stem cells, Pluripotency, Reprogramming

## Abstract

**Background:**

Transcription factor-mediated reprogramming can efficiently convert differentiated cells into induced pluripotent stem cells (iPSCs). Furthermore, many cell types have been shown to be amenable to reprogramming into iPSCs, such as neural stem cells, hematopoietic progenitor and stem cells (HPC/HSCs). However, the mechanisms related to the amenability of these cell types to be reprogrammed are still unknown.

**Methods:**

Herein, we attempt to elucidate the mechanisms of HPC/HSC reprogramming using the sequential reprogramming system that we have previously established.

**Results:**

We found that HPC/HSCs were amenable to transcription factor-mediated reprogramming, which yielded a high frequency of fully reprogrammed HPC/HSC-iPSCs. Genome-wide gene expression analyses revealed select down-regulated tumor suppressor and mesenchymal genes as well as up-regulated oncogenes in HPC/HSCs compared with mouse embryonic fibroblasts (MEFs), indicating that these genes may play important roles during the reprogramming of HPC/HSCs. Additional studies provided insights into the contribution of select tumor suppressor genes (*p21*, *Ink4a* and *Arf*) and an epithelial-to-mesenchymal transition (EMT) factor (*Snail1*) to the reprogramming process of HPC/HSCs.

**Conclusions:**

Our findings demonstrate that HPC/HSCs carry unique cellular characteristics, which determine the amenability of HPC/HSCs to be reprogrammed into high-quality iPSCs.

**Electronic supplementary material:**

The online version of this article (doi:10.1186/s13287-016-0298-z) contains supplementary material, which is available to authorized users.

## Background

The ectopic expression of a set of transcription factors, including Oct4, Sox2, Klf4, and c-Myc (OSKM), has been shown to convert somatic cells into induced pluripotent stem cells (iPSCs) [1]. The generation of iPSCs has tremendous therapeutic potential for making patient-specific iPSCs available [2–4]. To date, many types of cells, including embryonic fibroblasts, hepatocytes, gastric epithelial cells, adult tail-tip fibroblasts (TTFs), pancreatic cells, neural stem cells, B lymphocytes, T lymphocytes, and trophoblast stem cells, have been successfully converted into iPSCs [5–11]. In addition, iPSCs are indistinguishable from embryonic stem cells (ESCs) with respect to global transcription and epigenetic modifications and can achieve pluripotency. These findings have led to enthusiasm for conducting proof-of-principle safety evaluations for the therapeutic use of iPSCs [12–15]. The availability of high-quality iPSCs is a prerequisite for their potential therapeutic use.

During the reprogramming process, the epigenetic transition from starting cells to pluripotent cells can be achieved when cells reach an acquired pluripotency state [16]. Accumulating evidence suggests that the dynamics of nuclear reprogramming may be diverse; a typical example can be found in the reprogramming of B lymphocytes [8, 9]. Many studies have established that substitutes for the traditional four reprogramming factors, now known as “Yamanaka factors”, can be used to induce pluripotency [17, 18], indicating the complexity of the versatile reprogramming process. Likewise, many developmental events, such as the mesenchymal-to-epithelial transition (MET), have also been found to coincide with the nuclear reprogramming process [19]. The *Ink4a*/*Arf* locus, which encodes three tumor suppressor genes (p16^Ink4a^ and p19^Arf^ from *Cdkn2a* and p15^Ink4b^ from *Cdkn2b*), was found to be a barrier to reprogramming [20, 21], further emphasizing the relationship between immortalization and reprogramming. Furthermore, a negative correlation has been revealed between excessive cell proliferation and reprogramming efficiency in human somatic cells [22]. Therefore, understanding the molecular events of reprogramming in different cell types to elucidate the core circuit of nuclear reprogramming may allow more effective interventions for the reprogramming of cells.

Hematopoietic cells belong to a well-defined population of cells that have been rigorously defined based on the expression of cluster of differentiation (CD) molecules. These cells have been used widely in nuclear transfer-mediated and transcription factor-mediated cell reprogramming studies [10, 23]. One of the most notable breakthroughs in nuclear transfer-mediated reprogramming was carried out in terminally differentiated B and T lymphocytes [24], supporting the notion that the presence of residual adult stem cells in tissues does not account for the observed successful reprogramming of mammalian somatic cells [25]. Compared with somatic cells, the epigenetic state of neural stem cells has been proven amenable to nuclear reprogramming [7]. Additionally, hematopoietic progenitor and stem cells (HPC/HSCs) were found to be susceptible to transcription factor-mediated reprogramming [10]. However, the mechanisms related to the amenability of HPC/HSCs to be reprogrammed remain unknown.

Herein, we attempt to determine the mechanisms related to the amenability of HPC/HSCs to be reprogrammed by testing the potential of HPC/HSCs to be reprogrammed into high-quality iPSCs using our previously established sequential reprogramming system [26] and performing genome-wide gene expression analyses to determine the underlying events correlated with HPC/HSC reprogramming. Our results demonstrated that the downregulation of select tumor suppressor genes and an epithelial-to-mesenchymal transition (EMT) factor as well as the upregulation of oncogenes in HPC/HSCs undergo a unique reprogramming process through which HPC/HSCs are amenable to be reprogrammed into iPSCs. Further studies showed that tumor suppressor genes (*p21*, *Ink4a*, and *Arf*) and an EMT factor (*Snai1 1*) participated in the reprogramming of HPC/HSCs, in which independent ectopic activation of *p21*, *Ink4a*, *Arf*, and *Snail1* along with OSKM in HPC/HSCs decreased the reprogramming efficiency.

## Methods

### Animal welfare

The protocols of all animal experiments were approved by the Animal Care and Use Committee of the National Institute of Biological Sciences, Beijing, China. All animal procedures were performed according to the National Institute of Biological Sciences Guide for the Care and Use of Laboratory Animals.

### Isolation of HPC/HSCs

HPC/HSCs were isolated from tetraploid-complementation (4N) mice derived from mouse embryonic fibroblasts (MEFs) with a 129S2/Sv genetic background and a Rosa26-M2rtTA transgene [27]. In the isolation procedure, the 4N mice were euthanized, after which the tibia and femur were dissected from both legs and maintained in ice-cold PBE (phosphate-buffered saline (PBS) containing 0.5 % bovine serum albumin and 2 mM ethylenediamine tetraacetic acid). The muscles were removed from the bones using sharp surgical scissors; a 5 ml syringe containing ice-cold PBE was then inserted into one end of the bone, and the bone marrow was extruded into a 5 ml tube. After thorough mixing of the cell suspension, the cells were passed through a 70 μm nylon mesh filter into a fresh 5 ml tube to remove any cell clumps. The cell suspension was centrifuged at 300 × *g* for 10 minutes at 4 °C, the supernatant was discarded, and the cell pellet was resuspended in 80 μl PBE per 10^8^ total cells. Then, 20 μl of CD117 MicroBeads (Miltenyi, Bergisch Gladbach, Germany) was added to the cell suspension and incubated on ice for 15 minutes. The cells were washed twice with PBE in a final volume of 500 μl. Finally, the cell suspension was transferred to a PBE-pretreated MS column (Miltenyi, Bergisch Gladbach, Germany) under a magnetic field (MACS; Miltenyi, Bergisch Gladbach, Germany), and the magnetically labeled cells were flushed into PBE. The nucleated cells were centrifuged at 500 × *g* for 10 minutes.

### Flow cytometry

HSC/HPCs isolated by MACS were incubated with APC-CD117 (c-kit; eBioscience) and FITC-CD45.2 (eBioscience, San Diego, CA) and analyzed using LSR II (BD Biosciences, San Jose, CA) as described previously [28]. Flow cytometric analysis was performed for the cell proliferation rate using BD Pharmingen™ BrdU Flow Kits (BD Biosciences, San Jose, CA) according to the manufacturer’s instructions.

### Generation of HPC/HSC-iPSCs and cell culture

The generation of HPC/HSC-iPSCs was performed under the sequential reprogramming system we established [26]. In detail, 5 × 10^4^ HPC/HSCs were transferred to 3.5 cm dishes with ES medium containing 50 ng/ml murine stem cell factor (SCF; Peprotech, Rocky Hill, NJ), 10 ng/ml murine interleukin (IL)-3 (Peprotech, Rocky Hill, NJ), and 10 ng/ml murine IL-6 (Peprotech, Rocky Hill, NJ). Twenty-four hours later, the medium was replaced with ES medium containing 1 μg/ml doxycycline (Dox; Sigma, St. Louis, MO) to induce the expression of OSKM under the regulation of tetracycline response elements (TRE). Dox was removed on day 14. Two days after the withdrawal of Dox, ESC-like colonies were picked and passaged three days later to yield HPC/HSC-iPSCs. All ESCs and iPSCs were cultured on mitomycin C-treated (Sigma, St. Louis, MO) MEFs in ES medium, which consisted of Dulbecco modified Eagle’s medium (DMEM; Invitrogen, Carlsbad, CA) supplemented with 15 % fetal bovine serum (FBS; Hyclone, South Logan, Utah), 1 mM l-glutamine (Invitrogen, Carlsbad, CA), 0.1 mM β-mercaptoethanol (Invitrogen, Carlsbad, CA), 1 % nonessential amino acid (Invitrogen, Carlsbad, CA), and 1000 U/ml leukemia inhibitory factor (LIF; Millipore, Darmstadt, Germany).

### Quantitative PCR

We extracted mRNA using TRIzol (Invitrogen, Carlsbad, CA) and reverse-transcribed the mRNA using M-MLV reverse transcriptase (Promega, Madison, WI). Quantitative PCR (Q-PCR) was carried out with SYBR Green-based PCR Master Mix (Takara, Shiga, Japan). A total volume of 20 μl containing 10 μl SYBR Green-based PCR Master Mix, 0.2 mM dNTP, 0.2 μl forward primer (10 mM), 0.2 μl reverse primer (10 mM), and 0.2 μl dye II was mixed and plated for gene expression analyses using the relative quantitation (RQ) of gene expression of the Applied Biosystems 7500 Fast Real-Time PCR System (Thermo Fisher Scientific, Waltham, MA) in accordance with the manufacturer’s instructions. One independent experiment contained three replicates of both targeted genes and inner control. The results of three independent experiments in duplicate were averaged to calculate the mean value of every gene. Relative expression levels of target genes in each cell line were normalized to the level of their endogenous *Gapdh*. Paired Student’s *t* tests were performed to assess the statistical difference. The significant standard was set as: fold-change >2; *P* <0.05. The primer pairs for real-time PCR are summarized in Additional file 1: Table S1.

### Alkaline phosphatase and immunofluorescence staining

Alkaline phosphatase (AP) staining was performed using the Alkaline Phosphatase Detection Kit (Millipore, Darmstadt, Germany) according to the manufacturer’s instructions. Immunofluorescence staining was performed as described previously [29].

### Teratoma formation

For the teratoma formation assay, iPSCs suspended in PBS were injected subcutaneously into the forelimbs of SCID mice. SCID mice were sacrificed 3 or 4 weeks after the injection to collect the tumors, which were further dissected for hematoxylin and eosin (H & E) staining to identify the three germ layers.

### Bisulfite genomic sequencing

Bisulfite genomic sequencing was conducted in triplicate to analyze the DNA methylation of *Pou5f1* and *Nanog* as described previously [29]. The bisulfite PCR primer pairs are summarized in Additional file 1: Table S1. The amplified PCR products were cloned into a vector using the pEASYTM-T5 Zero cloning kit (TransGen, Beijing, China) and were sequenced by Invitrogen and SangonBiotech (Sangon, Shanghai, China).

### Generation of chimera and 4N mice

To generate chimera (2N) mice, 10–15 iPSCs were microinjected into eight-cell stage ICR embryos using a piezo-actuated microinjection pipette. The reconstructed embryonic day 2.5 embryos were then transplanted into the uteri of pseudo-pregnant mice. Tetraploid complementation was similarly performed using piezo-actuated microinjection. The two-cell stage ICR embryos were first electrofused into tetraploid embryos and cultured to blastocysts. Then, 10–15 iPSCs were injected into the cavum of the tetraploid blastocysts, which were then transplanted into the uteri of pseudo-pregnant mice. A cesarean section was performed at embryonic day 19.5, and the resultant pups were fostered by lactating ICR mothers.

### Simple sequence length polymorphism

Primers for simple sequence length polymorphism (SSLP) were selected according to the Mouse Genome Informatics website (http://www.informatics.jax.org) and performed as reported previously [26, 30].

### Western blot

Whole cell extracts were prepared using RIPA buffer and ultrasonic extraction, resolved on SDS-PAGE gels, and transferred to polyvinylidene fluoride membranes. Specific proteins were analyzed using anti-CDKN2A/p16INK4a (Abcam, Cambridge, UK), anti-CDKN2A/p19ARF (Abcam, Cambridge, UK), anti-SNAI1 (Millipore, Darmstadt, Germany), and anti-Gapdh (Sigma, St. Louis, MO). Enhanced chemiluminescence peroxidase-labeled anti-mouse, rabbit or goat antibodies (Amersham, Pittsburgh, PA) were used for further detection.

### Gene construction and transient and stable transfection

The cDNAs of *Ink4a, Arf*, *Ink4b*, *p21*, and *Snail1* were cloned separately into the TetO-FUW plasmid, and the reconstructed plasmids were transfected into 293T cells along with lentivirus packaging plasmids ps-PAX-2 and pMD2G. Viral supernatants were harvested 48 hours after transfection, filtered through a 0.45 μm filter (Millipore, Darmstadt, Germany), and concentrated by centrifugation. HPC/HSCs from 4N mice were incubated with virus resuspended in ES medium containing SCF, IL-3, and IL-6 for 24 hours; the medium was then replaced with ES medium containing 1 μg/ml Dox to access the reprogramming efficiency of specific candidate genes.

### RNA sequencing

Total RNA was isolated from cell pellets using TRIzol (Invitrogen, Carlsbad, CA). The RNA integrity was confirmed with a minimum RNA integrity number of 8 using 2100 Bioanalyzer (Agilent Technologies, Santa Clara, CA). The mRNA was enriched using oligo(dT) magnetic beads and sheared to create short fragments of ~200 base pairs. cDNA was then synthesized using random hexamer primers and purified using a PCR product extraction kit (Qiagen, Germany). Finally, the sequencing primers linked to the cDNA fragments were isolated by gel electrophoresis and enriched by PCR amplification to construct the library for sequencing. Single-end sequencing was applied to RNA sequencing at the Beijing Genomics Institute using the HiSeq™ 2000 system developed by Illumina (Illumina, San Diego, CA). The RNA sequencing reads were mapped to the mouse genome using Tophat (v1.3.3) and the Ensembl genome annotation (Mus_musculus.NCBIM37.64.gtf) with the default parameters [31]. The fragments per kilobase of exon per million fragments mapped (FPKM) for each gene were calculated using Cufflinks (v1.2.0) [32]. Hierarchical clustering was presented as mean ± standard deviation (SD) based on two biological replicates to describe the relationship among samples.

### Statistical analyses

The SD was used to assess biological significance.

### Accession numbers

The genome-wide gene expression data reported in this article were deposited in Gene Expression Omnibus [GEO:GSE36294].

## Results

### Derivation of high-quality iPSCs from HPC/HSCs

**Fig. 1:**
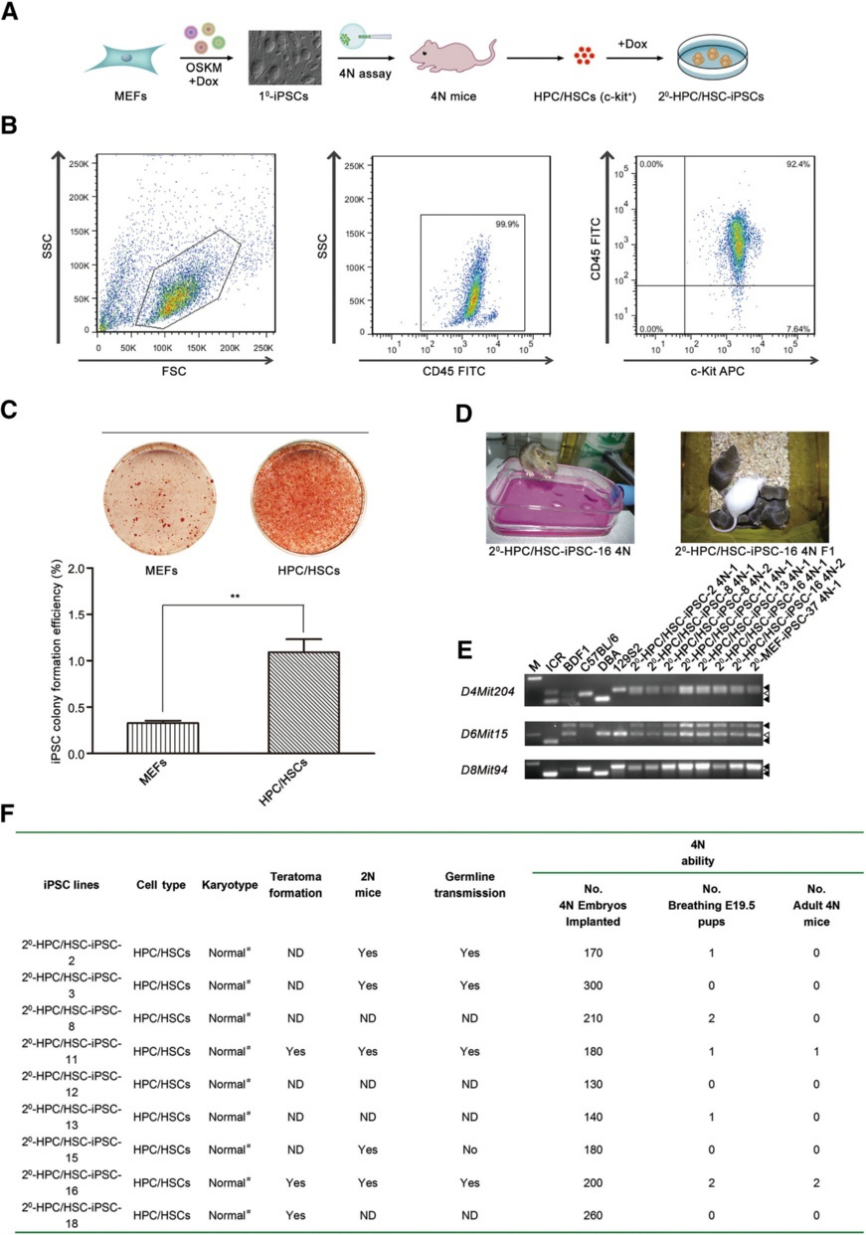
Transcription factor-mediated reprogramming of HPC/HSCs results in high-quality iPSCs with full pluripotency. **a** Schematic showing the generation of iPSCs from HPC/HSCs. **b** Flow cytometry analysis of the purity of HPC/HSCs isolated by MACS. *First column*, cells were isolated based on size, as indicated by side scatter (*SSC*) and forward scatter (*FSC*). *Second column*, CD45-positive cells were further selected. *Third column*, c-Kit and CD45 double-positive cells were isolated. **c** The reprogramming efficiency of HPC/HSCs was analyzed using AP staining. MEFs were used as the control (*n* = 3 measurements). Error bars indicate the SD. **P <0.01, unpaired *t* test. **d** Viable 4N mice and the offspring (F1) of 4N mice derived from HPC/HSC-iPSCs with the indicated cell line. **e** Genetic characterization of the 4N mice using SSLP analysis. **f** Summary of the derivation of mice from HPC/HSC-iPSCs. ^#^Karyotype was considered normal when greater than 80 %. *Dox* doxycycline, *E19.5* embryonic day 19.5, *HPC/HSC* hematopoietic progenitor and stem cell, *iPSC* induced pluripotent stem cell, *MEF* mouse embryonic fibroblast, *2N* chimera, *4N* tetraploid complementation, *ND* not determined, *OSKM* Oct4, Sox2, Klf4, and c-Myc. *APC* Allophycocyanin, *FITC* Fluorescein isothiocyanate

To determine the reprogramming potential of HPC/HSCs, our sequential reprogramming system was used to generate the genetically identical iPSCs [26, 27] (Fig. 1a). HPC/HSCs were isolated from 4N mice derived from 1^0^-MEF-iPSCs using MACS MicroBeads technology with a purity of approximately 92 % when analyzed by flow cytometry (Fig. 1b). The characteristics of the HPC/HSCs were verified by analyzing the expression of HPC/HSC-specific genes (Additional file 2: Figure S1A). Exposure of the freshly sorted HPC/HSCs to Dox and the addition of SCF, IL-3, and IL-6 resulted in re-expression of the four transcription factors (*Oct4*, *Sox2*, *Klf4*, and *c-Myc*) and rapid proliferation. The suspended HPC/HSCs adhered to the bottom of the culture dishes 3 days later, and the AP-positive ESC-like colonies emerged approximately 9 days after induction, much sooner than the control MEFs (Fig. 1c, top). The average reprogramming efficiency of HPC/HSCs was 1.1 % when measured by AP staining, approximately threefold greater than MEFs (Fig. 1c, bottom). Two days following the withdrawal of Dox, transgene-independent HPC/HSC-iPSC lines were established from day 16 ESC-like colonies that displayed typical expression patterns of pluripotency-related genes (*Oct4*, *Sox2*, and *SSEA-1*; Additional file 2: Figure S1B). In addition, the global expression patterns of HPC/HSC-iPSCs were indistinguishable from those of ESCs (Additional file 2: Figure S1C). Bisulfite genomic sequencing results showed that demethylation of *Pou5f1* and *Nanog* occurred in HPC/HSC-iPSCs (Additional file 2: Figure S1D). Notably, Q-PCR results showed that the exogenous OSKM expressions were silenced in HPC/HSC-iPSCs, which were well maintained under the expression of endogenous pluripotency genes (Additional file 3: Figure S2A). The in vivo differentiation potential of HPC/HSC-iPSCs was further confirmed by teratoma formation assays and three typical embryonic germ layers were observed (Additional file 3: Figure S2B). The birth of reconstructed 2N mice further validated the chimeric and germline transmission potential of HPC/HSC-iPSCs (Additional file 3: Figure S2C).

To stringently evaluate the pluripotency state of HPC/HSC-iPSCs, tetraploid complementation was performed on the HPC/HSC-iPSCs and viable 4N mice were ultimately generated (Fig. 1d). Healthy offspring were produced after the adult 4N mice were mated with female ICR mice (Fig. 1d). To confirm the genetic background of the 4N mice, SSLP analyses were performed. The SSLP results indicated that the 4N mice were indeed produced from HPC/HSC-iPSCs and that they had a 129/Sv × M2rtTA genetic background (Fig. 1e). To the best of our knowledge, this is the first demonstration that viable, fertile 4N mice can be generated from HPC/HSC-derived iPSCs.

Interestingly, a large proportion (5/9) of HPC/HSC-iPSC lines gave rise to 4N mice (Fig. 1f and Additional file 3: Figure S2C). Based on published results, the average ratio of 4N competent iPSCs never exceeds 40 % [14, 33]. To exclude the possibility that the sequential reprogramming system utilized here resulted in a large proportion of high-quality iPSCs, skin fibroblasts (SFs) and TTFs from the same 4N mouse as the HPC/HSCs were used to generate SF-iPSCs and TTF-iPSCs, respectively, and tetraploid complementation was performed to test the pluripotency of the derived cell lines. SF-iPSCs were found to achieve a full pluripotency state with low proportion (1/6; Additional file 4: Table S2). However, none of the TTF-iPSC lines (0/4) supported the full development of 4N mice (Additional file 4: Table S2). These results indicate that HPC/HSCs are more amenable to reprogramming into high-quality iPSCs than somatic cells.

### Genome signatures of HPC/HSCs correlated with accelerated reprogramming

**Fig. 2:**
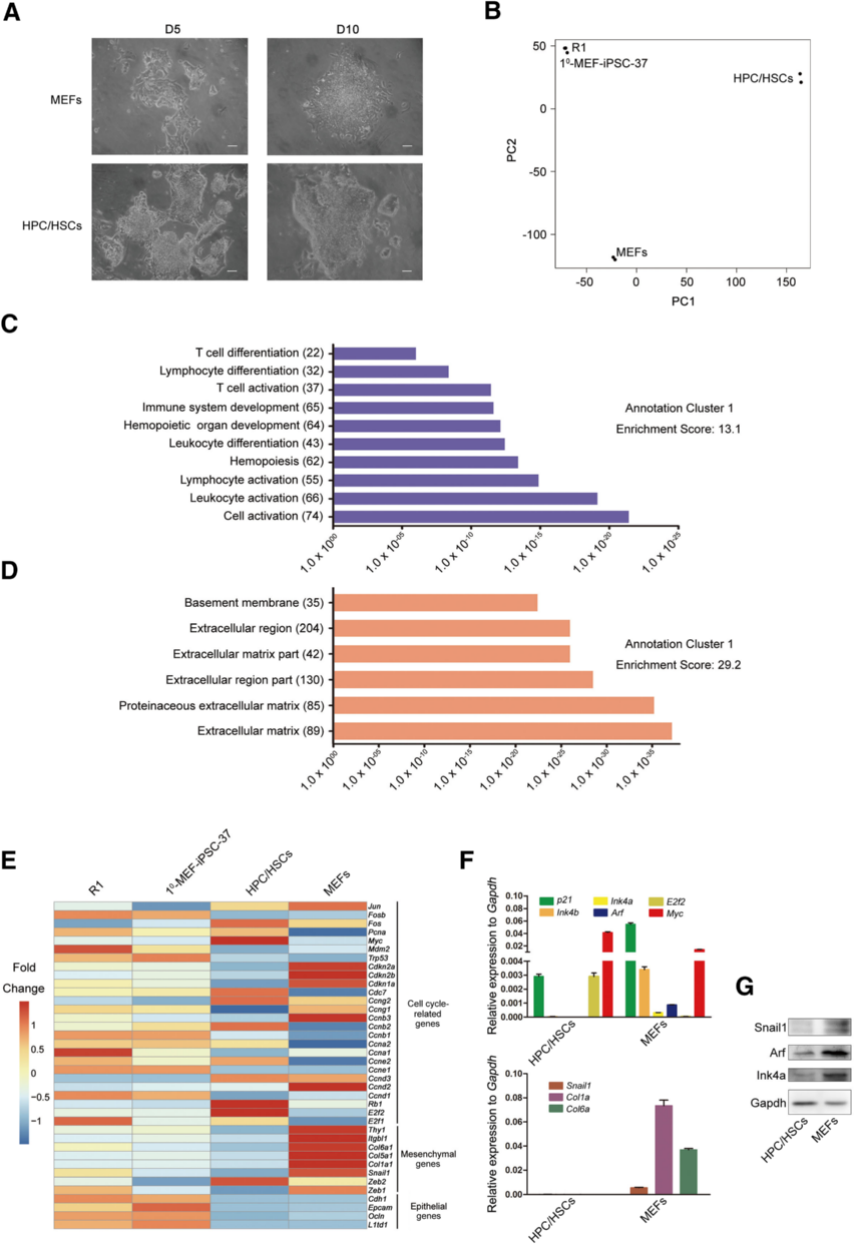
Genome-wide signature analyses reveal the unique characteristics related to HPC/HSC reprogramming. **a** Morphology differences during the reprogramming process of HPC/HSCs and MEFs. Scale bar, 100 μm. **b** Principle component analysis of global gene expression patterns in HPC/HSCs, MEFs, R1 ESCs, and iPSCs. The analysis of each cell line was based on two biological replicates. **c** Gene ontology (GO) of highly expressed genes in HPCs/HSCs. **d** GO of highly expressed genes in MEFs. **c**, **d** *X* axis depicts the *P* value. **e** Gene expression analyses of mesenchymal genes, epithelial genes, and cell cycle-related genes in HPC/HSCs and MEFs. The cluster analysis is presented as the mean ± SD based on two biological replicates. **f** Comparison of expression analyses of indicated genes in HPC/HSCs and MEFs (*n* = 3 measurements). Error bars indicate the SD. **g** Western blot comparing the levels of the indicated genes in HPC/HSCs and in MEFs. *D* day, *HPC/HSC* hematopoietic progenitor and stem cell, *iPSC* induced pluripotent stem cell, *MEF* mouse embryonic fibroblast

As already mentioned, HPC/HSCs are amenable to reprogramming into high-quality iPSCs, suggesting that the reprogramming process may be different in HPC/HSCs compared with MEFs. Concerning the morphology change of HPC/HSCs during the reprogramming process, precolonies were observed 3 days post reprogramming which are similar to the control MEFs (Additional file 5: Figure S3A). However, more compact colonies, which are an indication of naive pluripotency, were derived from HPC/HSCs in the middle and late stages of reprogramming when compared with MEFs (Fig. 2a). However, the underlying mechanisms of HPC/HSC reprogramming remain unclear.

To gain insight into the intrinsic events related to HPC/HSC reprogramming, genome-wide gene expression analyses were performed in HPC/HSCs using MEFs and pluripotent stem cells as controls. First, we compared the global gene expression patterns from HPC/HSCs, MEFs, R1(ESCs), and iPSCs (1^0^-MEF-iPSC-37). Principle component analysis (PCA) showed that HPC/HSCs and MEFs demonstrated gene expression patterns that differed from those of pluripotent stem cells, indicating that dramatic cell fate transitions occurred during the process of reprogramming (Fig. 2b). Next, HPC/HSC-specific and MEF-specific genes were subjected to gene ontology (GO) analysis; the results showed that highly expressed genes in HPC/HSCs mainly correlated with functions related to hematopoiesis (Fig. 2c). In MEFs, genes that were highly expressed were associated with extracellular matrix functions (Fig. 2d).

Previous studies have shown that epithelial cells can be reprogrammed with a higher efficiency than MEFs and that the induction of MET enhances reprogramming efficiency [19, 34]. Moreover, ultrafast cycling cell populations were found to be privileged adopters of the pluripotency state, suggesting a role for the cell cycle in reprogramming [35]. To investigate whether these two events also affected the reprogramming of HPC/HSCs, mesenchymal genes, epithelial genes, and cell cycle-related genes were analyzed in HPC/HSCs, MEFs, ESCs, and iPSCs. We found that the expression levels of mesenchymal genes (*Zeb1*, *Snail1*, *Col1a1*, *Col5a1*, *Col6a1*, *Itgbl1*, and *Thy1*) were downregulated in HPC/HSCs compared with MEFs, while epithelial genes (*L1td1*, *Ocln*, *Epcam*, and *Cdh1*) demonstrated no changes (Fig. 2e), suggesting that HPC/HSCs undergoing reprogramming might require fewer steps to switch off the MET program compared with MEFs. Flow cytometric analysis of 5-bromo-2′-deoxyuridine (BrdU) incorporation was performed on HPC/HSCs to assess cell proliferation following the first 48 hours after reprogramming using MEFs as the control. We found a higher number of S-phase (BrdU^+^) cells in HPC/HSCs compared with MEFs, indicating a rapid doubling of HPC/HSCs in the first 48 hours after reprogramming (Additional file 5: Figure S3B, C). Consistently, several tumor suppressor genes among the cell cycle-related genes, including *Cdkn1a*, *Cdkn2a*, *Cdkn2b*, and *Rb1*, showed lower expression patterns in HPC/HSCs than in MEFs, whereas oncogenes, such as *Myc* and *E2f2*, were more highly expressed in HPC/HSCs (Fig. 2e). Q-PCR results confirmed differential expression patterns for *p21* (encoded by *Cdkn1a*), *Ink4b* (encoded by *Cdkn2b*), *Ink4a* (encoded by *Cdkn2b*), *Arf* (encoded by *Cdkn2b*), *E2f2*, *Myc*, *Snail1*, *Col1a*, and *Col6a* between HPC/HSCs and MEFs (*t* test, *P* <0.05) (Fig. 2f). Western blot further confirmed the differential expression patterns of *Snail1*, *Arf*, and *Ink4a* (Fig. 2g) between HPC/HSCs and MEFs. These results indicate the potential role of these genes in HPC/HSC reprogramming.

### The participation of select tumor suppressor genes and an EMT factor in HPC/HSC reprogramming

**Fig. 3:**
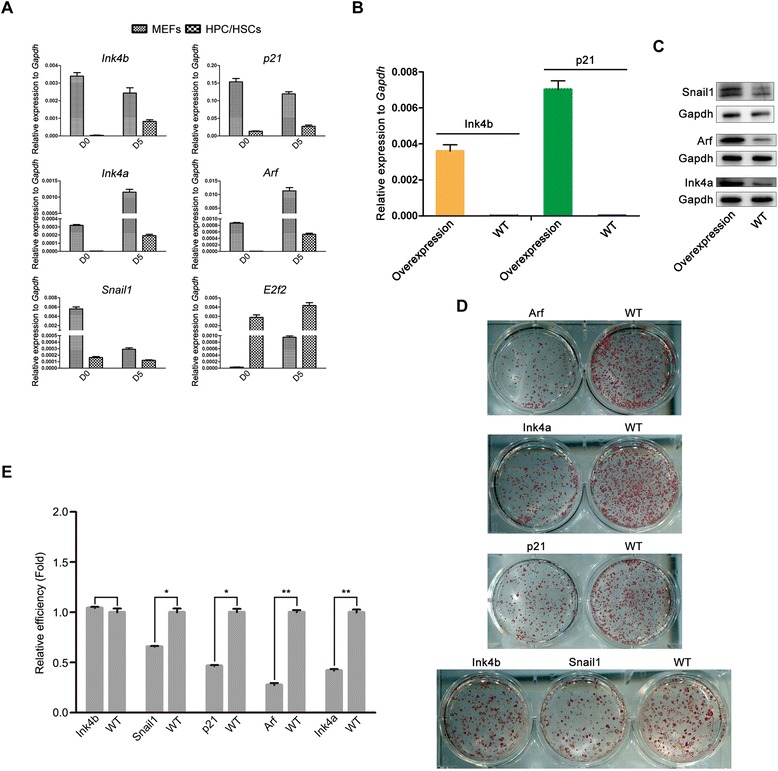
Participation of select tumor suppressor genes and an EMT factor in HPC/HSC reprogramming. **a** Kinetics of expression of indicated genes in the OSKM-mediated reprogramming of HPC/HSCs and MEFs (*n* = 3 measurements). Error bars indicate the SD. **b**, **c** Ectopic activation of indicated genes during the reprogramming process of HPC/HSCs. **d** AP staining results for ectopic activation of *p21*, *Ink4b*, *Ink4a*, *Arf*, and *Snail1* in the OSKM-mediated reprogramming of HPC/HSCs. **e** Reprogramming efficiencies of the indicated genotypes relative to wild-type (*WT*) in HPC/HSCs (*n* = 3 measurements). Error bars indicate the SD. *P <0.05, **P <0.01, unpaired *t* test. *D* day, *HPC/HSC* hematopoietic progenitor and stem cell, *MEF* mouse embryonic fibroblast

To elucidate the mechanisms underlying HPC/HSC reprogramming, the functions of select cell cycle-related genes (*p21*, *Ink4b*, *Ink4a*, *Arf*, and *E2f2*) and an EMT factor during reprogramming were studied. The kinetics of *p21*, *Ink4b*, *Ink4a*, *Arf*, *E2f2*, and *Snail1* expression during the reprogramming process of HPC/HSCs and MEFs were therefore analyzed for their potential functions. We found that the tumor suppressor genes *p21*, *Ink4b*, *Ink4a*, and *Arf*, as well as the EMT factor *Snail1*, showed lower expression in the intermediate HPC/HSCs than in the intermediate MEFs (*t* test, *P* <0.05), whereas the oncogene *E2f2* showed an opposite pattern (*t* test, *P* <0.05), indicating the potential participation of these genes in HPC/HSC reprogramming (Fig. 3a).

Independent ectopic activation of *p21* (*t* test, *P* <0.05), *Ink4b* (*t* test, *P* <0.05), *Ink4a*, *Arf*, and *Snail1*, along with OSKM, was performed to characterize the functions of these genes in HPC/HSC reprogramming (Fig. 3b, c). The results showed that the overexpression of *p21*, *Ink4a*, and *Arf* decreased the reprogramming efficiency of HPC/HSCs, but the overexpression of *Ink4b* exhibited no effect (Fig. 3d, e). Our observed roles of *p21*, *Ink4a*, and *Arf* in HPC/HSC reprogramming were consistent with previous reports [20, 21], suggesting that immortalization is indispensable for the accelerated reprogramming of HPC/HSCs. Similar to the tumor suppressor gene results, the overexpression of *Snail1* also decreased the efficiency of HPC/HSC reprogramming (Fig. 3d, e), which is in accordance with a previous report [19]. Taken together, these results indicate that select tumor suppressor genes and an EMT factor participate in the reprogramming of HPC/HSCs.

**Fig. 4:**
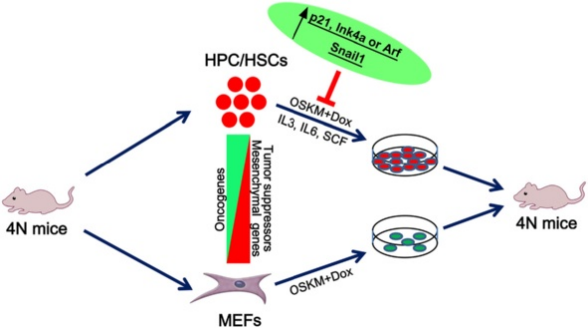
Schematic illustration of the participation of select tumor suppressor genes (*p21*, *Ink4a*, and *Arf*) and an EMT factor (*Snail1*) in the amenability of HPC/HSC reprogramming. Select tumor suppressor and mesenchymal genes were downregulated in HPC/HSCs and select oncogenes were upregulated in HPC/HSCs compared with MEFs, indicating that these genes may play important roles in the reprogramming of HPC/HSCs. The downregulation of tumor suppressor genes (*p21*, *Ink4a*, and *Arf*) and *Snail1* in HPC/HSCs triggers the amenability of HPC/HSCs to OSKM-mediated reprogramming. Independent ectopic activation of *p21*, *Ink4a*, *Arf*, and *Snail1* along with OSKM decreases the efficiency of HPC/HSC reprogramming. *Dox* doxycycline, *HPC/HSC* hematopoietic progenitor and stem cell, *IL* interleukin, *MEF* mouse embryonic fibroblast, *4N* tetraploid complementation, *OSKM* Oct4, Sox2, Klf4, and c-Myc, *SCF* stem cell factor

Collectively, the work presented here showed that the reprogramming of HPC/HSCs results in a high frequency of high-quality iPSCs with full pluripotency. Genome-wide analyses showed that the downregulation of select tumor suppressor genes and an EMT factor and the upregulation of select oncogenes occur in HPC/HSCs, suggesting that these factors are potentially involved in the amenability of HPC/HSCs to be reprogrammed. Further exploration demonstrated that select tumor suppressor genes (*p21*, *Ink4a*, and *Arf*) and an EMT factor (*Snail1*) participate in HPC/HSC reprogramming, in which independent ectopic activation of *p21*, *Ink4a*, *Arf*, and *Snail1* along with OSKM decreased the reprogramming efficiency of HPC/HSCs (Fig. 4).

## Discussion

In the present study, the mechanisms of reprogramming HPC/HSCs into high-quality iPSCs were investigated by a combination of techniques, including genome-wide analysis. To our knowledge, this study is the first to demonstrate that HPC/HSCs are amenable to be reprogrammed into high-quality iPSCs with full pluripotency at a high frequency. Through genome-wide analysis, we selected several candidates and further verified their potential contribution to HSC/HPC reprogramming. Furthermore, we discovered that select tumor suppressor genes (*p21*, *Ink4a*, and *Arf*) and an EMT factor (*Snail1*) participated in HPC/HSC reprogramming, suggesting that both immortalization and EMT transition are indispensable for HPC/HSC reprogramming.

The quality of iPSCs determines the biosafety of cell transplantation-based regenerative medicine. Many strategies have been used to improve the quality of iPSCs [9, 14]. A previous study has shown an association between differentiation state and the reprogramming efficiency of iPSCs [10]; however, the mechanisms correlated with the amenability of HPC/HSCs to reprogram into iPSCs have not been rigorously addressed. Moreover, it is of significance to test whether increased reprogramming efficiency can result in high-quality iPSCs with full pluripotency. Our finding that a large proportion of HPC/HSC-iPSCs show full pluripotency is comparable with previous results which used the overexpression of *Zscan4* during reprogramming [36]. The presence of a large proportion of HPC/HSC-iPSCs with full pluripotency in our study demonstrates the susceptibility of HPC/HSCs to reprogramming and, reciprocally, defines the notion that different cell types face distinct barriers to achieve pluripotency. Further elucidation of the mechanisms underlying somatic stem and progenitor cell reprogramming will undoubtedly shed light on our understanding of the 4N competency of iPSCs.

The cellular states of starting cells have been shown to have a great impact on the cellular reprogramming process [7]. Furthermore, the acquisition of immortalization is found to be a crucial and rate-limiting step in the establishment of pluripotency [21]; in particular, the abrogation of Trp53 eliminates the roadblock in iPSC cellular reprogramming [37, 38]. In addition, the acquisition of immortalization through the suppression of the *Ink4a*/*Arf* locus can accelerate iPSC reprogramming [20, 21], which may help the cell bypass reprogramming barriers represented by the typical P53 and Rb pathways. In addition, recent data have shown that an ultrafast cycling cell population pre-existing among hematopoietic progenitors displays privileged induced reprogramming activity [35]. Furthermore, considerable evidence suggests that the EMT factor *Snail1* exerts dual effects which depend on the induction of EMT in the early stage of reprogramming and that of MET in the late stage of reprogramming [39, 40]. Similarly, we show in the present study that HPC/HSCs which demonstrate the downregulation of select tumor suppressor genes and an EMT factor and the upregulation of oncogenes could achieve pluripotency with high efficiency. Gene function analyses showed that select tumor suppressor genes and an EMT factor play important roles in HPC/HSC reprogramming, a finding that is consistent with previous reports [19, 20].

However, the efficiency of induced reprogramming observed in the present study was lower than in previous reports [10, 35]. This inconsistency may have resulted from the different origins of the cells used in these studies as well as from differences in the technical details of the reprogramming assay. The HPC/HSCs that were used in the present study included myeloid progenitors and HSCs. The major difference between these two cell types is the cycling time, with HSCs being slow cycling. In addition, the HPC/HSCs used here were cultured in ES medium with SCF, IL-3, and IL-6 for 24 hours before exposure to Dox, a method that may preferentially impact the proliferation of the ultrafast cycling population among the bulk cells. Nevertheless, under the reprogramming conditions used in the current study, high-quality iPSCs were obtained from HPC/HSCs. Further investigation of the reprogramming of HPC/HSCs will not only provide more information on the molecular barriers to reprogramming but might also provide alternatives that make it possible to overcome these inherent barriers to achieving pluripotency. Finally, it will be interesting to examine whether high-quality iPSCs can be generated from progenitor cells derived from other somatic tissues.

## Conclusions

HPC/HSCs that exhibited a downregulation of select tumor suppressor and mesenchymal genes and an upregulation of select oncogenes were amenable to transcription factor-mediated reprogramming, which yielded a high frequency of fully reprogrammed HPC/HSC-iPSCs. Additional studies provided insights into the contribution of select tumor suppressor genes (*p21*, *Ink4a*, and *Arf*) and an EMT factor (*Snail1*) to the amenability of HPC/HSC reprogramming.

## Additional files

Additional file 1: is Table S1 presenting the list of primers. (DOC 68 kb) Additional file 2: is Figure S1 showing cellular characteristics and HPC/HSC reprogramming. **A** Identification of HPC/HSCs using Q-PCR to detect genes (*Ikzf1*, *Lyl1*, and *Myb*) specifically expressed by HPC/HSCs. MEFs, adipose progenitor cells (*APCs*), and R1 ESCs were used as controls (*n* = 3 measurements). Error bars indicate the SD. **B** Expression of pluripotency-related markers (Pou5f1, Sox2, and SSEA-1) detected by immunofluorescence. DNA was stained with 4′,6-diamidino-2-phenylindole (*DAPI*). **C** HPC/HSC-iPSCs were indistinguishable from MEF-iPSCs at the level of global gene expression. *Cor* Pearson correlation coefficient, *X, Y* log expression value. **D** Bisulfite genomic sequencing of *Pou5f1* and *Nanog* in HPC/HSC-iPSCs. R1 ESCs were used as the control. *Black circle* methylated CpG; *white circle* unmethylated CpG. (TIF 1727 kb) Additional file 3: is Figure S2 showing silencing of OSKM factors and the pluripotency state of HPC/HSC-iPSCs. **A** Q-PCR was used to detect the gene expression levels of *Pou5f1*, *Sox2*, *Klf4*, and *c-Myc* in the indicated cell lines. *Gray column* total; *white column* endogenous; *red column* exogenous. Relative expression levels of these genes (*Y* axis) in each cell line were first normalized to the level of their endogenous *Gapdh*, and then the amount was normalized to R1 (*n* = 3 measurements). Error bars indicate the SD. **B**) Hematoxylin and eosin (*HE*) staining of teratomas derived from iPSCs. Ectoderm, lamellar corpuscle (×200); mesoderm, cartilage (×400); and endoderm, columnar epithelium and gland (×400). **C** viable 2N and 4N mice derived from HPC/HSC-iPSCs with the indicated cell line. The placenta of each newborn 4N mouse is displayed on the left of the 4N pup. (TIF 9159 kb) Additional file 4: is Table S2 presenting characteristics of the SF-iPSCs and TTF-iPSCs. (DOC 28 kb) Additional file 5: is Figure S3 showing the cell morphology change and cell proliferation rate of HPC/HSCs compared with MEFs. A The cell morphology changes in the early reprogramming process of HPC/HSCs and MEFs. Scale bar, 100 μm. B Flow cytometric analysis of BrdU/7-Aminoactinomycin D (*7-AAD*) incorporation was performed on HPC/HSCs during the first 48 hours after reprogramming to assess cell proliferation. MEFs were used as the control. C Frequencies of cell cycle phases in the early intermediate cells (48 hours after reprogramming) of HPC/HSC and MEF populations (*n* = 3 measurements). Error bars indicate the SD. **P* <0.05, ***P* <0.01, unpaired *t* test. (TIF 2724 kb)

## Abbreviations

AP: Alkaline phosphatase
BrdU: 5-Bromo-2′-deoxyuridine
CD: Cluster of differentiation
DMEM: Dulbecco modified Eagle’s medium
Dox: Doxycycline
EMT: Epithelial-to-mesenchymal transition
ESC: Embryonic stem cell
FBS: Fetal bovine serum
FPKM: Fragments per kilobase of exon per million fragments mapped
GO: Gene ontology
H & E: Hematoxylin and eosin
HPC/HSC: Hematopoietic progenitor and stem cell
IL: Interleukin
iPSC: Induced pluripotent stem cell
LIF: Leukemia inhibitory factor
MEF: Mouse embryonic fibroblast
MET: Mesenchymal-to-epithelial transition
2N: Chimera
4N: Tetraploid complementation
OSKM: Oct4, Sox2, Klf4, and c-Myc
PBE: Phosphate-buffered saline containing 0.5 % bovine serum albumin and 2 mM ethylenediamine tetraacetic acid
PBS: Phosphate-buffered saline
PCA: Principle component analysis
Q-PCR: quantitative PCR
RQ: Relative quantitation
SCF: Stem cell factor
SD: Standard deviation
SF: Skin fibroblast
SSLP: Simple sequence length polymorphism
TRE: Tetracycline response elements
TTF: Tail-tip fibroblast

## References

[1] Takahashi K, Yamanaka S (2006). Induction of pluripotent stem cells from mouse embryonic and adult fibroblast cultures by defined factors. Cell.

[2] Park IH, Arora N, Huo H, Maherali N, Ahfeldt T, Shimamura A (2008). Disease-specific induced pluripotent stem cells. Cell.

[3] Soldner F, Hockemeyer D, Beard C, Gao Q, Bell GW, Cook EG (2009). Parkinson's disease patient-derived induced pluripotent stem cells free of viral reprogramming factors. Cell.

[4] Takebe T, Sekine K, Enomura M, Koike H, Kimura M, Ogaeri T (2013). Vascularized and functional human liver from an iPSC-derived organ bud transplant. Nature.

[5] Aoi T, Yae K, Nakagawa M, Ichisaka T, Okita K, Takahashi K (2008). Generation of pluripotent stem cells from adult mouse liver and stomach cells. Science.

[6] Zhao XY, Li W, Lv Z, Liu L, Tong M, Hai T (2010). Viable fertile mice generated from fully pluripotent iPS cells derived from adult somatic cells. Stem Cell Rev.

[7] Kim JB, Zaehres H, Wu G, Gentile L, Ko K, Sebastiano V (2008). Pluripotent stem cells induced from adult neural stem cells by reprogramming with two factors. Nature.

[8] Hanna J, Markoulaki S, Schorderet P, Carey BW, Beard C, Wernig M (2008). Direct reprogramming of terminally differentiated mature B lymphocytes to pluripotency. Cell.

[9] Stadtfeld M, Apostolou E, Ferrari F, Choi J, Walsh RM, Chen T (2012). Ascorbic acid prevents loss of Dlk1-Dio3 imprinting and facilitates generation of all-iPS cell mice from terminally differentiated B cells. Nat Genet.

[10] Eminli S, Foudi A, Stadtfeld M, Maherali N, Ahfeldt T, Mostoslavsky G (2009). Differentiation stage determines potential of hematopoietic cells for reprogramming into induced pluripotent stem cells. Nat Genet.

[11] Wu T, Wang H, He J, Kang L, Jiang Y, Liu J (2011). Reprogramming of trophoblast stem cells into pluripotent stem cells by Oct4. Stem Cells.

[12] Guenther MG, Frampton GM, Soldner F, Hockemeyer D, Mitalipova M, Jaenisch R (2010). Chromatin structure and gene expression programs of human embryonic and induced pluripotent stem cells. Cell Stem Cell.

[13] Kang L, Wang J, Zhang Y, Kou Z, Gao S (2009). iPS cells can support full-term development of tetraploid blastocyst-complemented embryos. Cell Stem Cell.

[14] Zhao XY, Li W, Lv Z, Liu L, Tong M, Hai T (2009). iPS cells produce viable mice through tetraploid complementation. Nature.

[15] Boland MJ, Hazen JL, Nazor KL, Rodriguez AR, Gifford W, Martin G (2009). Adult mice generated from induced pluripotent stem cells. Nature.

[16] Hochedlinger K, Jaenisch R (2006). Nuclear reprogramming and pluripotency. Nature.

[17] Heng JC, Feng B, Han J, Jiang J, Kraus P, Ng JH (2010). The nuclear receptor Nr5a2 can replace Oct4 in the reprogramming of murine somatic cells to pluripotent cells. Cell Stem Cell.

[18] Gao Y, Chen J, Li K, Wu T, Huang B, Liu W (2013). Replacement of Oct4 by Tet1 during iPSC induction reveals an important role of DNA methylation and hydroxymethylation in reprogramming. Cell Stem Cell.

[19] Li R, Liang J, Ni S, Zhou T, Qing X, Li H (2010). A mesenchymal-to-epithelial transition initiates and is required for the nuclear reprogramming of mouse fibroblasts. Cell Stem Cell.

[20] Li H, Collado M, Villasante A, Strati K, Ortega S, Canamero M (2009). The Ink4/Arf locus is a barrier for iPS cell reprogramming. Nature.

[21] Utikal J, Polo JM, Stadtfeld M, Maherali N, Kulalert W, Walsh RM (2009). Immortalization eliminates a roadblock during cellular reprogramming into iPS cells. Nature.

[22] Gupta MK, Teo AK, Rao TN, Bhatt S, Kleinridders A, Shirakawa J (2015). Excessive cellular proliferation negatively impacts reprogramming efficiency of human fibroblasts. Stem Cells Transl Med.

[23] Sung LY, Gao S, Shen H, Yu H, Song Y, Smith SL (2006). Differentiated cells are more efficient than adult stem cells for cloning by somatic cell nuclear transfer. Nat Genet.

[24] Hochedlinger K, Jaenisch R (2002). Monoclonal mice generated by nuclear transfer from mature B and T donor cells. Nature.

[25] Wilmut I, Schnieke AE, McWhir J, Kind AJ, Campbell KH (1997). Viable offspring derived from fetal and adult mammalian cells. Nature.

[26] Gao S, Zheng C, Chang G, Liu W, Kou X, Tan K (2015). Unique features of mutations revealed by sequentially reprogrammed induced pluripotent stem cells. Nat Commun.

[27] Brambrink T, Foreman R, Welstead GG, Lengner CJ, Wernig M, Suh H (2008). Sequential expression of pluripotency markers during direct reprogramming of mouse somatic cells. Cell Stem Cell.

[28] Liu Y, Cheng H, Gao S, Lu X, He F, Hu L (2014). Reprogramming of MLL-AF9 leukemia cells into pluripotent stem cells. Leukemia.

[29] Chang G, Miao YL, Zhang Y, Liu S, Kou Z, Ding J (2010). Linking incomplete reprogramming to the improved pluripotency of murine embryonal carcinoma cell-derived pluripotent stem cells. PLoS One.

[30] Chang G, Gao S, Hou X, Xu Z, Liu Y, Kang L (2014). High-throughput sequencing reveals the disruption of methylation of imprinted gene in induced pluripotent stem cells. Cell Res.

[31] Trapnell C, Pachter L, Salzberg SL (2009). TopHat: discovering splice junctions with RNA-Seq. Bioinformatics.

[32] Roberts A, Pimentel H, Trapnell C, Pachter L (2011). Identification of novel transcripts in annotated genomes using RNA-Seq. Bioinformatics.

[33] Kang L, Wu T, Tao Y, Yuan Y, He J, Zhang Y (2011). Viable mice produced from three-factor induced pluripotent stem (iPS) cells through tetraploid complementation. Cell Res.

[34] Aasen T, Raya A, Barrero MJ, Garreta E, Consiglio A, Gonzalez F (2008). Efficient and rapid generation of induced pluripotent stem cells from human keratinocytes. Nat Biotechnol.

[35] Guo S, Zi X, Schulz VP, Cheng J, Zhong M, Koochaki SH (2014). Nonstochastic reprogramming from a privileged somatic cell state. Cell.

[36] Jiang J, Lv W, Ye X, Wang L, Zhang M, Yang H (2013). Zscan4 promotes genomic stability during reprogramming and dramatically improves the quality of iPS cells as demonstrated by tetraploid complementation. Cell Res.

[37] Rasmussen MA, Holst B, Tumer Z, Johnsen MG, Zhou S, Stummann TC (2014). Transient p53 suppression increases reprogramming of human fibroblasts without affecting apoptosis and DNA damage. Stem Cell Reports.

[38] Kawamura T, Suzuki J, Wang YV, Menendez S, Morera LB, Raya A (2009). Linking the p53 tumour suppressor pathway to somatic cell reprogramming. Nature.

[39] Unternaehrer JJ, Zhao R, Kim K, Cesana M, Powers JT, Ratanasirintrawoot S (2014). The epithelial-mesenchymal transition factor SNAIL paradoxically enhances reprogramming. Stem Cell Reports.

[40] Liu X, Sun H, Qi J, Wang L, He S, Liu J (2013). Sequential introduction of reprogramming factors reveals a time-sensitive requirement for individual factors and a sequential EMT-MET mechanism for optimal reprogramming. Nat Cell Biol.

