# Supplementary material for: Genome-wide gene expression analyses reveal unique cellular characteristics related to the amenability of HPC/HSCs into high-quality induced pluripotent stem cells
Source: Stem Cell Res Ther. 2016 Mar 15;7:40. doi: 10.1186/s13287-016-0298-z (PMC4791787; doi:10.1186/s13287-016-0298-z)
Supplement: Additional file 1: — is Table S1 presenting the list of primers. (DOC 68 kb) [file 13287_2016_298_MOESM1_ESM.doc]

**Table S1. The list of primers**

| **Gene** | **Accession number** | **Sequence 5’→ 3’** | **PCR Sort** |
| --- | --- | --- | --- |
| *Ikzf1* | NM_001025597.1 | F: GAAAGGAGAGCCCCCCAGTCAG | Q-PCR |
|  |  | R: CGAGGCATCAAGCATTCGTAAA | Q-PCR |
| *Lyl1* | NM_008535.2 | F: CAGGCCAGAGCAGAGGTGGGTTC | Q-PCR |
|  |  | R: CTGTTGAGGAAGGGGTGAGGGTG | Q-PCR |
| *Myb* | NM_010848.3 | F: CCAGTCACGTTCCCTATCCTGTC | Q-PCR |
|  |  | R: TGGTAATGCCTGCTGTCCCTTCA | Q-PCR |
| *Ink4a* | NM_001040654.1 | F: CGTACCCCGATTCAGGTGAT | Q-PCR |
|  |  | R: TTGAGCAGAAGAGCTGCTACGT | Q-PCR |
| *Ink4b* | NM_007670.4 | F: AGATCCCAACGCCCTGAAC | Q-PCR |
|  |  | R: CCCATCATCATGACCTGGATT | Q-PCR |
| *Arf* | NM_009877.2 | F: GCCGCACCGGAATCCT | Q-PCR |
|  |  | R: TTGAGCAGAAGAGCTGCTACGT | Q-PCR |
| *p21* | NM_007669.4 | F: GTGGGTCTGACTCCAGCCC | Q-PCR |
|  |  | R: CCTTCTCGTGAGACGCTTAC | Q-PCR |
| *Snail1* | NM_011427.2 | F: GGCTGATGGAGTGCCTTTGT | Q-PCR |
|  |  | R: TGAGGGAGGTAGGGAAGTGG | Q-PCR |
| *Col1a* | NM_007742.3 | F: AGGGCGAGTGCTGTGCTTTC | Q-PCR |
|  |  | R: GGACCAGGAGGACCAGGAAGT | Q-PCR |
| *Col6a* | NM_009933.4 | F: TGTGGTGCAGACATTCAGGTAG | Q-PCR |
|  |  | R: CTTCAGAAAGCCGTCATCGAG | Q-PCR |
| *E2f2* | NM_177733.6 | F: CTTCGCTTTACACGCAGACG | Q-PCR |
|  |  | R: GGCTTGGCAAACCATCCAC | Q-PCR |
| *Pou5f1* | NM_013633.2 | Total F: AATGCCGTGAAGTTGGAGAAGG | Q-PCR |
|  |  | Total R: AAAGAGAACGCCCAGGGTGAGC | Q-PCR |
|  |  | Endo F: TCTTTCCACCAGGCCCCCGGCTC | Q-PCR |
|  |  | Endo R: TGCGGGCGGACATGGGGAGATCC | Q-PCR |
|  | TetO-FUW | F: AGTATTGAGTATTCCCAACGAG | Q-PCR |
|  |  | R: AGCGTATCCACATAGCGTAA | Q-PCR |
| *Sox2* | NM_011443.3 | Total F: CAGCATGATGCAGGAGCAGC | Q-PCR |
|  |  | Total R: CTGGAGTGGGAGGAAGAGGT | Q-PCR |
|  |  | Endo F: TAGAGCTAGACTCCGGGCGATGA | Q-PCR |
|  |  | Endo R: TTGCCTTAAACAAGACCACGAAA | Q-PCR |
|  | TetO-FUW | F: CATGACCAGCTCGCAGACC | Q-PCR |
|  |  | R: AGCGTATCCACATAGCGTAA | Q-PCR |
| *Klf4* | NM_004235.3 | Total F: CCAAAGAGGGGAAGAAGGTCG | Q-PCR |
|  |  | Total R: GTGCCTGGTCAGTTCATCGG | Q-PCR |
|  |  | Endo F: CCATCGGACCTACTTATCTGC | Q-PCR |
|  |  | Endo R: AAAACCTCAAACCAAAACCC | Q-PCR |
|  | TetO-FUW | F: GAACTGACCAGGCACTACCG | Q-PCR |
|  |  | R: AGCGTATCCACATAGCGTAA | Q-PCR |
| *Myc* | NM_010849.4 | Total F: GACTGTATGTGGAGCGGTTTC | Q-PCR |
|  |  | Total R: GCTGTCGTTGAGCGGGTAG | Q-PCR |
|  |  | Endo F: TGACCTAACTCGAGGAGGAGCTGGAATC | Q-PCR |
|  |  | Endo R: AGTTTGAGGCAGTTAAAATTATGGCTGAAGC | Q-PCR |
|  | TetO-FUW | F: ACGACAAGAGGCGGACAC | Q-PCR |
|  |  | R: AGCGTATCCACATAGCGTAA | Q-PCR |
| *Pou5f1* | NC_000083.5 | F1: GAGGATTGGAGGTGTAATGGTTGTT | Bisulfite PCR |
|  |  | R1: CTACTAACCCATCACCCCCACCTA | Bisulfite PCR |
|  |  | F2: TGGGTTGAAATATTGGGTTTATTT | Bisulfite PCR |
|  |  | R2: CTAAAACCAAATATCCAACCATA | Bisulfite PCR |
| *Nanog* | NC_000072.5 | F: AAGTATGGATTAATTTATTAAGGTAGTT | Bisulfite PCR |
|  |  | R1: AAAAAACCCACACTCATATCAATATA | Bisulfite PCR |
|  |  | R2: CAACCAAATCAACCTATCTAAAAA | Bisulfite PCR |
