# Supplementary figures and images for: Genome-wide gene expression analyses reveal unique cellular characteristics related to the amenability of HPC/HSCs into high-quality induced pluripotent stem cells
Source: Stem Cell Res Ther. 2016 Mar 15;7:40. doi: 10.1186/s13287-016-0298-z (PMC4791787; doi:10.1186/s13287-016-0298-z)

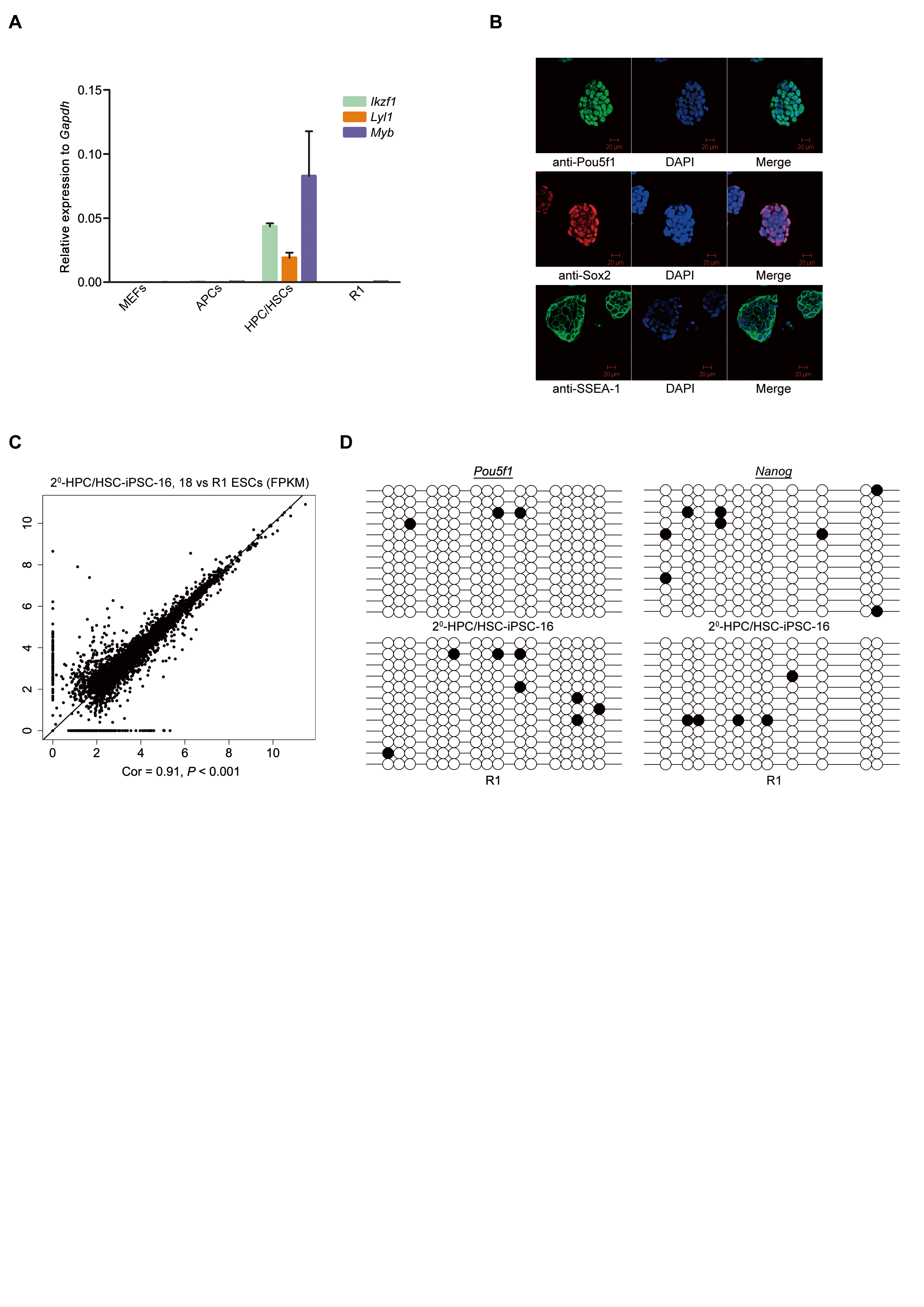

Supplement: Additional file 2: — is Figure S1 showing cellular characteristics and HPC/HSC reprogramming. A Identification of HPC/HSCs using Q-PCR to detect genes (Ikzf1, Lyl1, and Myb) specifically expressed by HPC/HSCs. MEFs, adipose progenitor cells (APCs), and R1 ESCs were used as controls (n = 3 measurements). Error bars indicate the SD. B Expression of pluripotency-related markers (Pou5f1, Sox2, and SSEA-1) detected by immunofluorescence. DNA was stained with 4′,6-diamidino-2-phenylindole (DAPI). C HPC/HSC-iPSCs were indistinguishable from MEF-iPSCs at the level of global gene expression. Cor Pearson correlation coefficient, X, Y log expression value. D Bisulfite genomic sequencing of Pou5f1 and Nanog in HPC/HSC-iPSCs. R1 ESCs were used as the control. Black circle methylated CpG; white circle unmethylated CpG. (TIF 1727 kb) [file 13287_2016_298_MOESM2_ESM.tif]

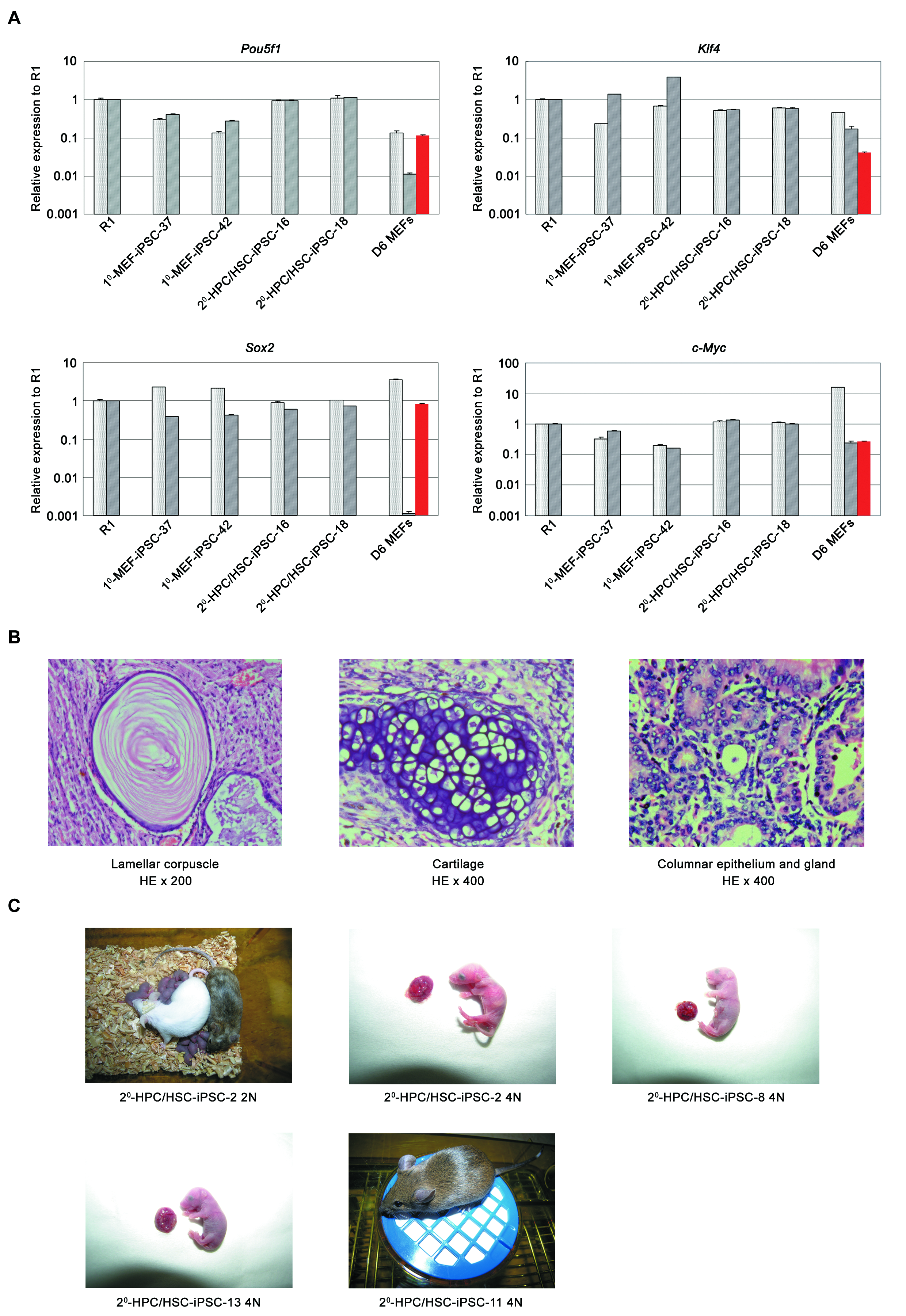

Supplement: Additional file 3: — is Figure S2 showing silencing of OSKM factors and the pluripotency state of HPC/HSC-iPSCs. A Q-PCR was used to detect the gene expression levels of Pou5f1, Sox2, Klf4, and c-Myc in the indicated cell lines. Gray column total; white column endogenous; red column exogenous. Relative expression levels of these genes (Y axis) in each cell line were first normalized to the level of their endogenous Gapdh, and then the amount was normalized to R1 (n = 3 measurements). Error bars indicate the SD. B) Hematoxylin and eosin (HE) staining of teratomas derived from iPSCs. Ectoderm, lamellar corpuscle (×200); mesoderm, cartilage (×400); and endoderm, columnar epithelium and gland (×400). C viable 2N and 4N mice derived from HPC/HSC-iPSCs with the indicated cell line. The placenta of each newborn 4N mouse is displayed on the left of the 4N pup. (TIF 9159 kb) [file 13287_2016_298_MOESM3_ESM.tif]

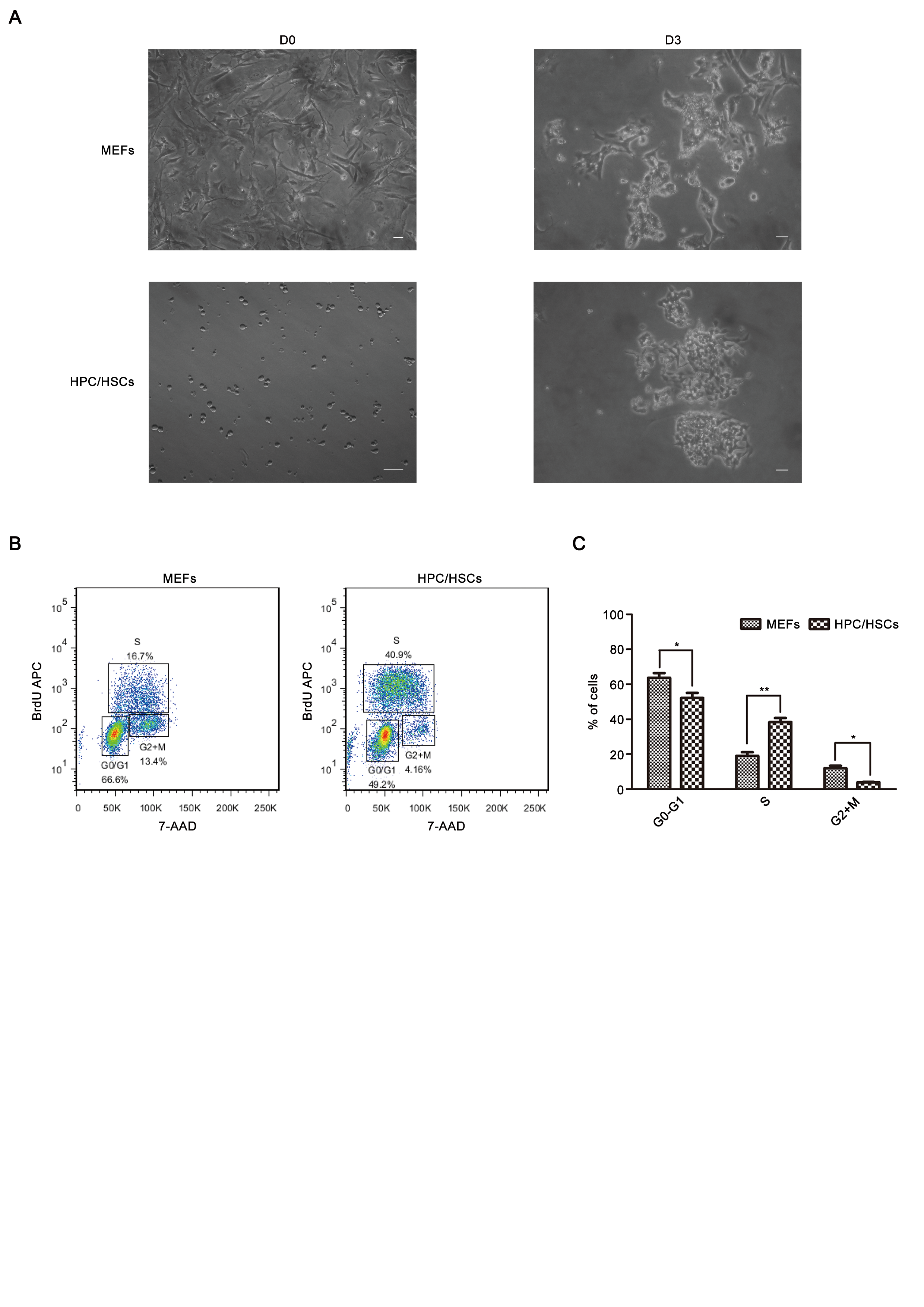

Supplement: Additional file 5: — is Figure S3 showing the cell morphology change and cell proliferation rate of HPC/HSCs compared with MEFs. A The cell morphology changes in the early reprogramming process of HPC/HSCs and MEFs. Scale bar, 100 μm. B Flow cytometric analysis of BrdU/7-Aminoactinomycin D (7-AAD) incorporation was performed on HPC/HSCs during the first 48 hours after reprogramming to assess cell proliferation. MEFs were used as the control. C Frequencies of cell cycle phases in the early intermediate cells (48 hours after reprogramming) of HPC/HSC and MEF populations (n = 3 measurements). Error bars indicate the SD. *P <0.05, **P <0.01, unpaired t test. (TIF 2724 kb) [file 13287_2016_298_MOESM5_ESM.tif]
