# Supplementary material for: Genome-wide gene expression analyses reveal unique cellular characteristics related to the amenability of HPC/HSCs into high-quality induced pluripotent stem cells
Source: Stem Cell Res Ther. 2016 Mar 15;7:40. doi: 10.1186/s13287-016-0298-z (PMC4791787; doi:10.1186/s13287-016-0298-z)
Supplement: Additional file 4: — is Table S2 presenting characteristics of the SF-iPSCs and TTF-iPSCs. (DOC 28 kb) [file 13287_2016_298_MOESM4_ESM.doc]

**Table S2. Characteristics of the skin fibroblast- and tail tip fibroblast-iPSCs**

| **iPSC lines** | **Cell type** | **Karyotype** | **Teratoma**  **formation** | **2N**  **mice** | **Germline**  **transmission** | **4N**  **ability** | | |
| --- | --- | --- | --- | --- | --- | --- | --- | --- |
|  |  |  |  |  |  | **No.**  **4N Embryos**  **Implanted** | **No.**  **Breathing E19.5**  **pups** | **No.**  **Adult 4N**  **mice** |
| 2^0^-SF-iPSC-1 | Skin Fibroblasts | Normal**^#^** | ND | ND | ND | 120 | 0 | 0 |
| 2^0^-SF-iPSC-4 | Skin Fibroblasts | Normal**^#^** | ND | ND | ND | 130 | 0 | 0 |
| 2^0^-SF-iPSC-6 | Skin Fibroblasts | Normal**^#^** | ND | ND | ND | 270 | 0 | 0 |
| 2^0^-SF-iPSC-7 | Skin Fibroblasts | Normal**^#^** | ND | ND | ND | 160 | 0 | 0 |
| 2^0^-SF-iPSC-10 | Skin Fibroblasts | Normal**^#^** | ND | ND | ND | 180 | 1 | 0 |
| 2^0^-SF-iPSC-11 | Skin Fibroblasts | Normal**^#^** | ND | ND | ND | 250 | 0 | 0 |
| 2^0^-TTF-iPSC-2 | Tail Tip Fibroblasts | Normal**^#^** | ND | ND | ND | 200 | 0 | 0 |
| 2^0^-TTF-iPSC-3 | Tail Tip Fibroblasts | Normal**^#^** | ND | ND | ND | 210 | 0 | 0 |
| 2^0^-TTF-iPSC-6 | Tail Tip Fibroblasts | Normal**^#^** | ND | ND | ND | 225 | 0 | 0 |
| 2^0^-TTF-iPSC-8 | Tail Tip Fibroblasts | Normal**^#^** | ND | ND | ND | 140 | 0 | 0 |

ND, not determined. #, karyotype was considered normal when greater than 80%.
